# Endothelial Progenitor Cells in the Pathogenesis of Idiopathic Pulmonary Fibrosis: An Evolving Concept

**DOI:** 10.1371/journal.pone.0053658

**Published:** 2013-01-14

**Authors:** Foteini Malli, Angela Koutsokera, Efrosini Paraskeva, Epaminondas Zakynthinos, Maria Papagianni, Dimosthenes Makris, Irene Tsilioni, Paschalis Adam Molyvdas, Konstantinos I. Gourgoulianis, Zoe Daniil

**Affiliations:** 1 Respiratory Medicine Department, School of Medicine, University of Thessaly, Larissa, Greece; 2 Department of Physiology, School of Medicine, University of Thessaly, Larissa, Greece; 3 Department of Critical Care Medicine, School of Medicine, University of Thessaly, Larissa, Greece; University of Pittsburgh, United States of America

## Abstract

**Background:**

Idiopathic pulmonary fibrosis (IPF) has been associated with abnormal vascular remodeling. Bone marrow derived endothelial progenitor cells (EPCs) are considered to possess lung tissue repair and vascular remodeling properties.

**Objectives:**

The study aimed to assess early EPCs levels and EPCs endogenous vascular endothelial growth factor (VEGF) expression in IPF. In order to examine alterations in the mobilization of EPCs from the bone marrow we measured plasma VEGF.

**Main Results:**

Twenty-three patients with IPF and fifteen healthy subjects were included. The number of early EPCs colonies was markedly reduced in IPF patients vs controls (6.00±6.49 vs 49.68±16.73, respectively, p<0.001). EPCs were further decreased in patients presenting systolic pulmonary arterial pressure (sPAP)≥35 mmHg. The number of colonies per well correlated negatively with P_(A-a)_O_2_ (r =  −0.750, p<0.001). Additionally, VEGF mRNA levels were significantly increased in IPF patients. There were no differences observed in VEGF plasma levels in IPF patients when compared to controls.

**Conclusions:**

The current data suggest that inadequate levels of early EPCs may potentially contribute to suppressed repair and recovery of the damaged pulmonary endothelium and thereby may drive the sequence of events in profibrogenic direction. Increased VEGFmRNA levels in the clinical context of IPF may represent a compensatory mechanism to overcome reduced EPCs levels.

## Introduction

Idiopathic pulmonary fibrosis (IPF) is a chronic, progressive fibrotic lung disease which is associated with lack of effective treatment and thus, poor survival [1], [2]. Despite extensive research, IPF pathogenesis remains unknown. Multiple mechanisms have been proposed to play a role in IPF pathogenesis including abnormal vascular repair and remodeling. Indeed, there is a body of evidence suggesting that in IPF, the impairment of repair reendothelization mechanisms following alveolar injury may lead to destruction in lung architecture and fibrosis [3]. Notably, failure of reendothelization may induce loss of the alveolar-capillary barrier integrity which might be considered as the point after which fibrosis may be inevitable.

Endothelial progenitor cells (EPCs) represent a subset of bone marrow-derived stem cells which may be essential in parenchymal repair and reconstitution of the damaged vascular bed [4], [5]. Two subtypes of EPCs exist. Late EPCs may differentiate into mature endothelial cells and repair injured blood vessels [6] while “early EPCs”(i.e. EPCs that grow into colony forming units (CFU) on fibronectin following 5–7 days culture) may have angiogenic potential by secreting cytokines such as VEGF and enhance the angiogenic process [7], [8]. The important role of EPCs in lung repair has been suggested from previous studies in animal models and humans [5], [9]. Notably, reduced EPCs numbers have been associated with persistent fibrotic changes, not due to IPF, in humans following lung injury [5]. In this respect, one might argue that EPCs might also play a pivotal role in IPF [8]. However, data in IPF patients are sparse to few reports [8].

We therefore aimed to see whether IPF patients present reduced circulating early EPCs compared to controls and whether this is associated with clinical indices of disease severity. Furthermore, we sought to investigate soluble VEGF serum levels and mRNA VEGF expression in order to provide further insight in the proangiogenic activity and mobilization of EPCs from the bone marrow.

## Methods

### Subjects

The present prospective case control study was conducted in the University Hospital of Larissa, Larissa, Greece. Patients were recruited by consecutive sampling from the respiratory outpatient clinic of the University Hospital of Larissa, Greece, between Jan 2008-September 2011. Controls were recruited during the same period from healthy volunteers. Patients fulfilled the following criteria: a)IPF diagnosis based on standard criteria [10], b)free of any treatment for IPF, c)absence of infection in 6 weeks prior to inclusion. The diagnosis was revised according to the new criteria for IPF [11] that were published during the preparation of the manuscript. All patients were classified as definite IPF. Exclusion criteria were: a)secondary causes of lung fibrosis (environmental or occupational exposure, drug toxicity, connective tissue disease, b)conditions that could affect EPCs levels, e.g. diseases associated with neo-vascularization (diabetes, cancer, ischemic disease, etc) treatment with statins and current smoking. Control group included subjects who were matched in terms of age and sex with patients and their medical history was free of lung or other known disease, were free of smoking and they received no drugs during the previous period while they had normal pulmonary function tests (PFTs). This study was approved by the University Hospital of Larissa ethics committee (5-10-2010). Written informed consent from all subjects was obtained.

All participants underwent clinical examination, PFTs (Bodyplethysmograph, Master-Screen Body, Viasys Healthcare, Germany) and arterial blood gas (ABGs) analysis (model 1630; Instrumentation Laboratories, Milan Italy). As some patients were under oxygen therapy we calculated alveolar to arterial (A-a) gradient for the assessment of their oxygenation, by the formula: P_(A–a)_O_2_ = (713×FiO_2_–1,25×PCO_2_)–P_a_O_2_. PFTs were performed by a technician who was blinded to patients medical history. Venous blood was collected, centrifuged at 1500g for 10 min at 4°C and plasma was collected and frozen at −80°C until measurements.

### Primary Cell Culture Assay for EPCs and RNA Isolation and Real Time q-PCR

Isolation of early EPCs was performed as previously reported [12]. Briefly, a 20 ml sample of venous blood was obtained in a heparinized tube from all participants and processed within 2 hours after collection. Peripheral blood mononuclear cells were isolated by Ficoll density centrifugation (Histopaque 1077, Sigma-Aldrich, Taufkirchen Germany), suspended in growth medium consisting of Medium 199 (GIBCO BRL Life Technologies) supplemented with 20 percent calf serum, penicillin (100 µg/ml) and streptomycin (100 µg/ml), and plated onto fibronectin-coated six-well plates (Biocoat BD Biosciences (5 million cells per well). After 48 hours nonadherent cells were collected and replated onto fibronectin-coated 24-well plates (1 million cells per well). Growth medium was changed every 3 days and the number of colonies was counted 7 days after the initial plating. Colonies were counted manually under an inverted microscope in a minimum of 4 wells. Colonies of early EPCs were characterized by a central cluster of rounded cells surrounded by multiple spindle-shaped thin flat cells. A central cluster of cells alone without associated emanating cells was not counted as a colony.

Although early EPCs may not be of endothelial origin as recent studies have suggested [8] we chose to use immunochemistry in order to characterize these cells as previously described [13]. Briefly, indirect immunostaining was performed with endothelial-specific antibodies directed against VEGF receptor 2 (Flk1(Flk-1, Santa Cruz Technology) and CD31 (R&D Systems, Mineapolis, USA). For control analysis, the primary antibodies were replaced by equal amounts of the corresponding normal rabbit or mouse IgG (CY3 conjugated). The control procedure revealed no appreciate staining.

For the assessment of VEGFmRNA expression by RT-PCR, EPCs were harvested after 7 days of initial plating. Total RNA was isolated by Trizol reagent (Invitrogen) according to the manufacturer’s protocol. Total RNA was reversed transcribed with the High capacity cDNA Reverse transcription kit (Applied Biosystems International). Quantitative RT-PCR was performed in a Miniopticon RT PCR system (BIORAD) using the IQ SYBR Green Supermix (BIORAD) and primers for human VEGF. The levels of mRNA were normalized to human beta actin mRNA. The VEGF mRNA was amplified using the following primers: VEGF forward 5′-CCCACTGAGGAGTCCAACATC-3′, VEGF reverse 5′-GGCCTT GGTGAGGTTTGATC-3′. Internal control β-actin mRNA was amplified using primers: Forward 5′-CCAACCGCGAGAAGATGA-3′ and Reverse 5′-CCAGAGGCGTACAGGGATAG. Amplification conditions were: 50°C for 2 minutes, 95°C for 10 minutes and then 95°C for 15 seconds, 60°C for 60 seconds for 40 cycles, followed by a melting curve analysis. Each sample was assayed in duplicate for both target and internal control and relative quantitative gene expression was calculated using the DDCT method and the relative expression software tool (REST) and presented as relative units.

### VEGF Assay

VEGF levels were measured with a commercially available enzyme immunosorbent assay kit (R&D Systems, Mineapolis, USA) according to the manufacturer’s protocol. The lower detection limit for plasma VEGF was 9 pg/ml.

### Transthorasic Echocardiography

Transthoracic echocardiogram was performed by physician (EZ) who was blinded to the patients disease severity and pulmonary function tests, within 7 days from the EPC isolation. M-mode, two-dimensional colour Doppler and Tissue Doppler imaging (TDI) were performed using standard methodology and commercially available equipment (GE Medical Systems-Vivid 3, Milwaukee, WI, USA, with a 1.5–3.6 MHz transducer).

Tricuspid regurgitant flow (TR) was identified by colour flow Doppler and the peak tricuspid regurgitant velocity was measured by continuous wave Doppler and transtricuspid gradient was assessed, based on the modified Bernoulli equation [14]. The highest velocity obtained from multiple views was used. Agitated saline was used to enhance suboptimal Doppler signals [15]. The diameter of the inferior vena cava and its respiratory variation were used to estimate right atrial pressure which was added to the transtricuspid gradient in order to estimate sPAP.

The mitral flow pattern was analyzed using the pulsed-wave Doppler technique, with the sample volume located between the tips of the mitral leaflets. TDI was analysed with sample volume located at the lateral site and the inerventricular septum of the mitral annulus in the four chamber view from the apical window. All results were the average of at least three different beats.

Mild or moderate TR was found in all patients allowing a quite good Doppler envelope. Although right ventricular (RV) function was not in exclusion criteria, all patients had fairly good systolic function without RV dilation; yet, the interventricular septum performed a normal movement i.e. without budging towards the left ventricle. All patients were in sinus rhythm; heart rate was less than 100/min during echo measurements. sPAP over 35 mmHg was used as a cut-off value for the presence of pulmonary hypertension (PH) [16], [17].

### Statistical Analysis

Data are presented as mean±SD or as median (25^th^ to 75^th^ percentile). Normal distribution was assessed by the Kolmogorov-Smirnov test. Comparison between patients and controls was performed with the use of Student’s *t* test or Mann-Whitney U-test according to variable distribution. Univariate correlations were performed by Pearson’s correlation coefficient or by Spearman’s correlation coefficient according to variable distribution. A p-value of <0.05 was considered to be statistically significant. Analysis was performed using the SPSS 16 statistical package (SPSS Chicago, IL).

## Results

The study population consisted of 23 IPF patients and 15 healthy volunteers. Baseline characteristics of participants are shown in Table 1. Mean(±SD) age (years) was 68.42(±6.25). All patients claimed dyspnea. Table 2 shows PFTs, TTE results and ABGs analysis. DL_CO_ was 42.50±18.36 mol/min/KPa.

**Table 1:** Demographic data and blood cell counts of the study population.

Parameter	Controls (n = 15)	IPF patients (n = 23)
Age (years)	65.5±3.51	68.42(±6.25).
Sex (M/F)	12/3	19/4
Smoking status (Current/Ex/Never smoker)	0/10/5	0/17/6
Blood cell counts		
RBC (10^6^µL^−1^)	5.19±0.21	5.00±0.49
WBC (10^3^µL^−1^)	7.62±1.95	8.01±1.56
Monocytes (µL^−1^)	610±124.23	632.16±189.45

Data are expressed as mean±SD or as percentages.

**Table 2:** Clinical characteristics and pulmonary function data of the study population.

Variable	Controls	IPF patients
FVC (%pred)	87.93±4.61	66.23±18.05*
DL_CO_ (%pred)	87.49±4.89	42.50±18.36 *
RV (%pred)	84.00±2.70	58.63±09.28*
TLC (%pred)	87.10±5.07	71.45±15.32*
PaO_2_ (mmHg)	–	81.8±9.46
PCO_2_ (mmHg)	–	40.07±3.99
P_(A-a)_O_2_ (mmHg)	–	20.35±8.84
sPAP (mmHg)	–	39.59±12.35

Data are expressed as mean±SD.

* p<0.05 as compared with controls.

### Cultures of EPCs

**Figure 1:**
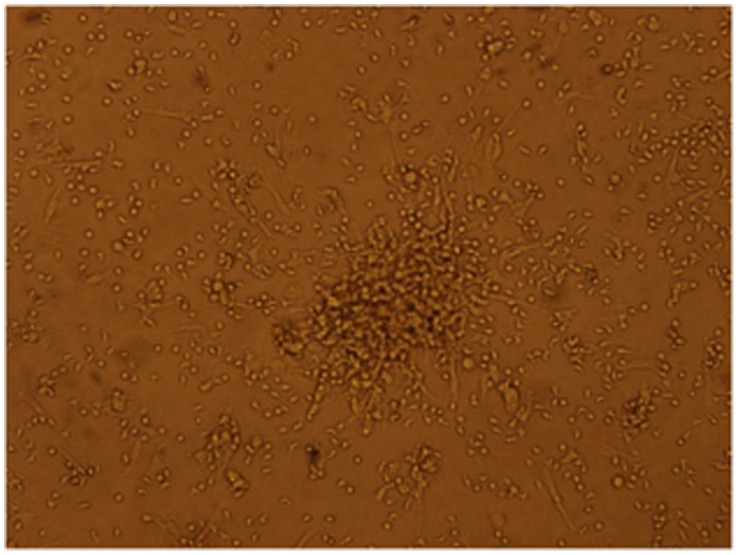
Representative phase contrast microscopy of EPCs colony from an IPF patient. Original magnification 10x.

**Figure 2:**
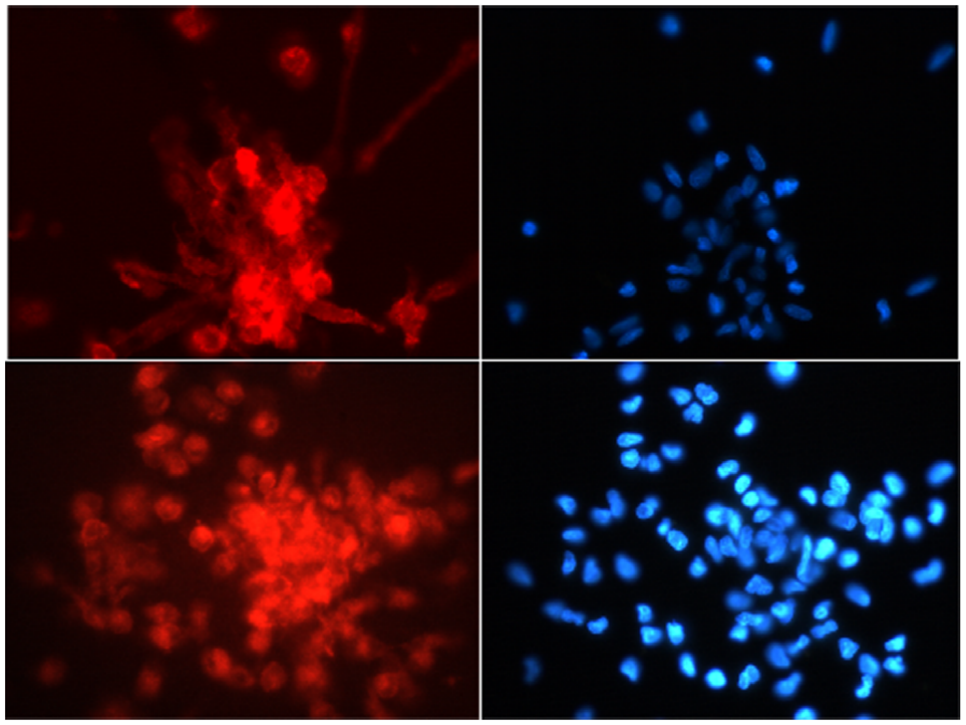
Reactivities for CD31 and Flk-1 are shown in the left column (upper left for CD31 and lower left for FlK-1, respectively); the corresponding DAPI-images of attached EPCs are shown in the right column. No staining was observed when the primary antibody was replaced by the corresponding control mouse and rabbit IgG, respectively (data not shown). Original magnification, 20x.

Morphologically, early EPCs colonies were characterized by a central cluster of rounded cells surrounded by multiple radiating thin flat cells. Representative microphotograph of an EPCs colony of an IPF patient is presented in Figure 1. Figure 2 presents indirect immunostaining with the use of endothelial-specific antibodies against VEGF receptor 2(Flk-1) and CD31 (Figure 2) was performed.

### EPCs Colonies

**Figure 3:**
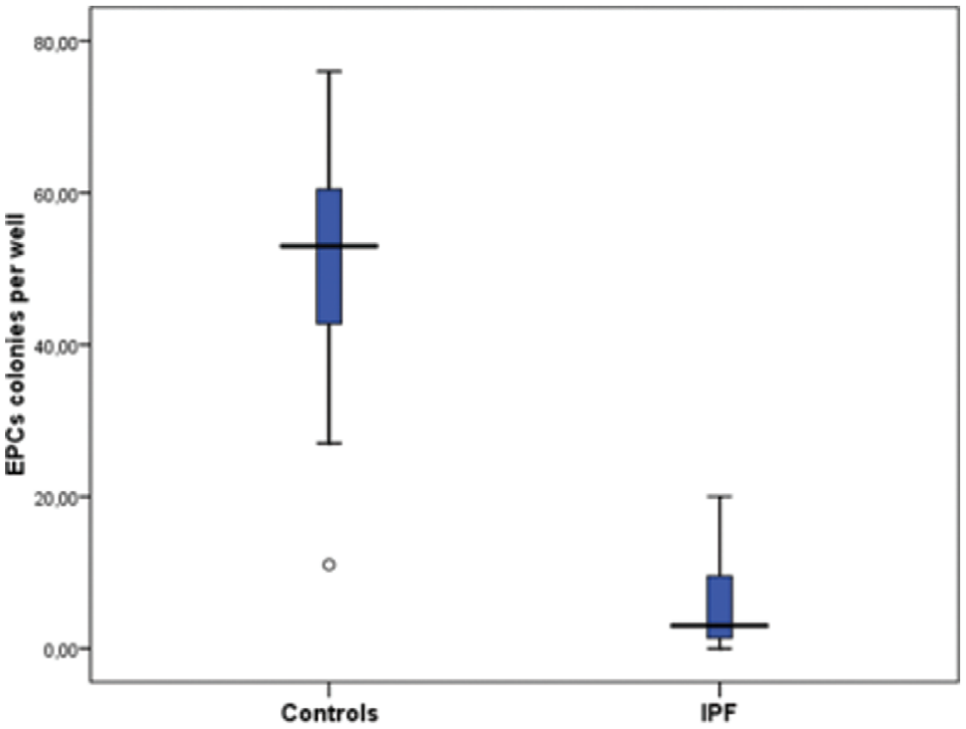
Number of EPCs colonies per well from control subjects and IPF patients (p<0.001).

**Figure 4:**
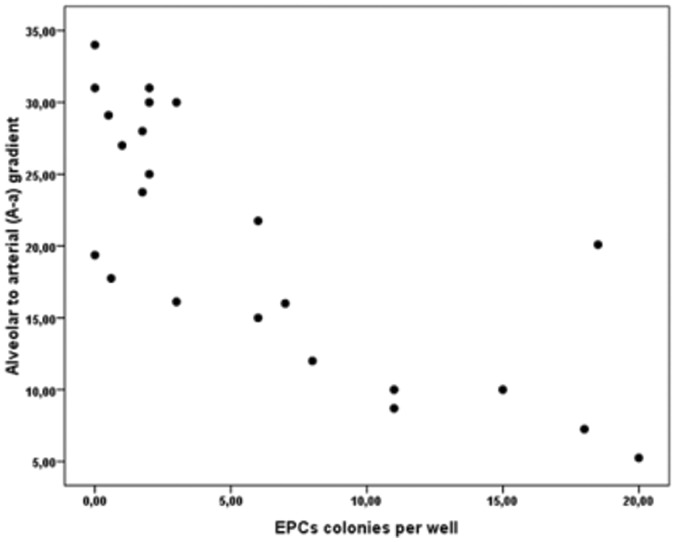
Relationship between the number of EPCs colonies per well and the alveolar to arterial gradient in the IPF patients (r =  −0.750, p<0.001).

**Figure 5:**
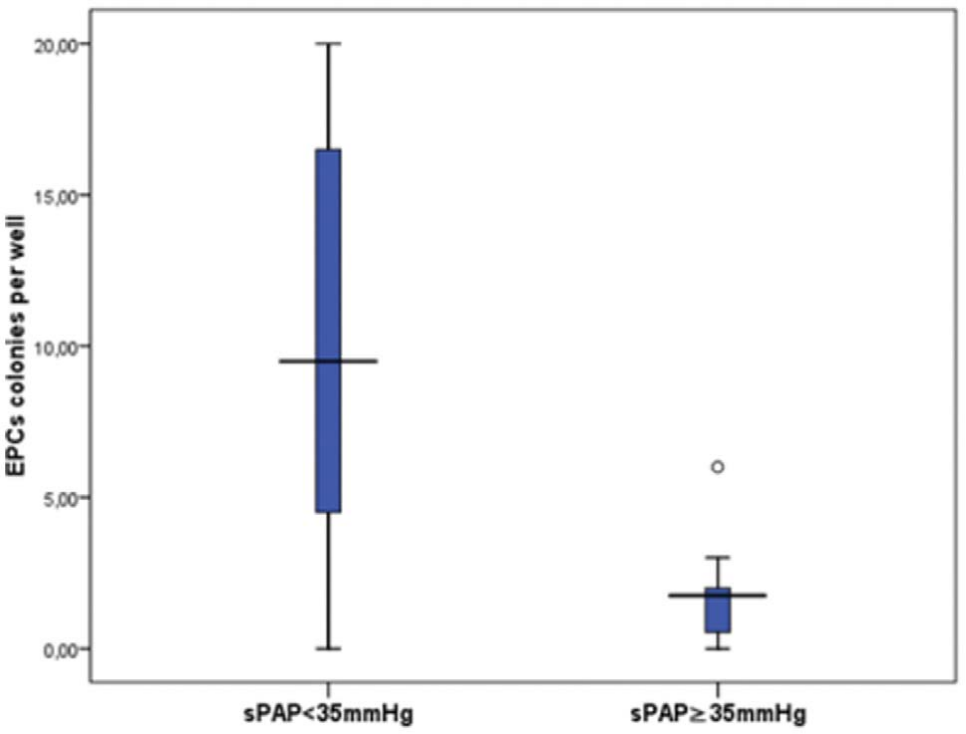
Number of EPCs colonies per well from IPF patients with sPAP<35 mmHg and sPAP≥35 mmHg (p = 0.001).

IPF patients exhibited statistically significantly decreased number of early EPCs CFU per well when compared to controls (6.00±6.49 vs 49.68±16.73, respectively, p<0.001), (Figure 3). The number of colonies per well correlated statistically significantly with P_(A-a)_O_2_ (R = −0.750, p<0.001) in IPF patients (Figure 4). When we evaluated EPCs CFU according to the degree of sPAP we found that IPF patients with sPAP≥35 mmHg had statistically significant reduced number of early EPCs colonies when compared with patients with sPAP<35 mmHg (1.69±1.71 vs 9.95±6.77, p = 0.001) (Figure 5).

No statistically significant relationship was observed between EPCs and the following parameters: age (r = 0.282, p = 0.40), FVC_%pred_ (r = 0.261, p = 0.466), DLCO_%pred_ (r = 0.389, p = 0.073), RV_%pred_ (r =  −0.235, p = 0.514), TLC_%pred_ (r =  −0.182, p = 0.614), PaO_2_ (r = 0.299, p = 0.345) and PCO_2_ (r = 0.298, p = 0.346).

### VEGF mRNA Expression in EPCs

**Figure 6:**
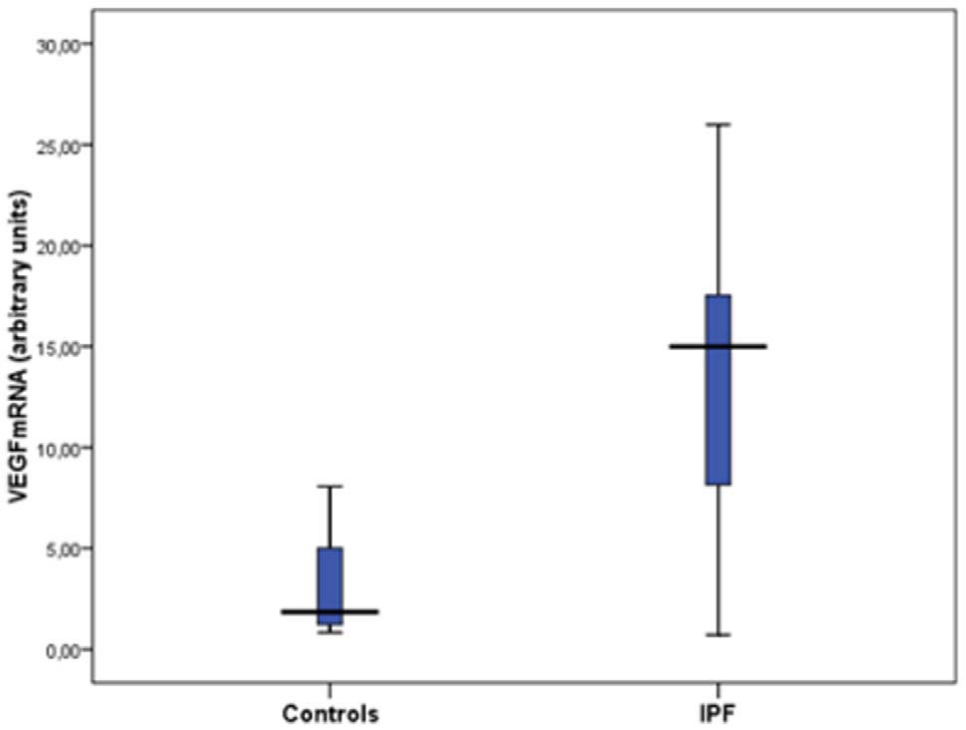
Endogenous VEGF expression in control subjects and IPF patients (p = 0.002).

Quantification of mRNA levels by real-time PCR showed a statistically significant difference in the relative VEGF expression between controls and IPF patients [2.99±2.42 vs 12.88±7.89 respectively, p = 0.002] (Figure 6).

### Systemic VEGF Concentrations

There were no significant differences observed in VEGF plasma levels in IPF patients when compared to controls [51.85(32.4–126.6) vs 42.18(25.34–56.59), respectively, p = 0.283].

## Discussion

In the present study we cultured early EPCs obtained from IPF subjects and healthy controls and we demonstrated that IPF patients presented significantly decreased EPCs colonies compared to controls. Our observation was more striking in IPF patients who presented signs of important cardiorespiratory physiologic deterioration (i.e. increased sPAP). Notably, this subgroup of IPF patients presented the most significant reduction in early EPCs. In addition, we observed increased endogenous VEGF expression in early EPCs derived from IPF patients which in turn may reflect a compensatory mechanism to overcome reduced EPCs levels and retain endothelial homeostasis in the IPF lung. In this respect, the findings of the present study underline potentially significant implications of early EPCs in IPF and might provide further insight in the understanding of the pathogenesis of the disease.

The role of EPCs in lung fibrotic processes -especially in lung repair following injury- has been underlined by previous studies [5]. EPCs can differentiate into mature endothelial cells and physically integrate into nascent blood vessels [6] while “early EPCs”(i.e. EPCs that grow into CFU on fibronectin following 5–7 days culture) enhance the angiogenic process by releasing growth factors. This is important in order to maintain normal endothelial function and repair after injury [4]. Autologous administration of early EPCs can preserve pulmonary endothelium and maintain the integrity of pulmonary alveolar-capillary barrier in animal models of acute lung injury suggesting that despite their limited proliferative ability they are able to induce endothelial sprouting [18]. In this respect, reduced numbers of EPCs may impair normal mechanisms of lung repair.

EPCs engraftment to the injured parenchyma attenuates lung injury by reestablishing the endothelial function and by preserving the integrity of the alveolar-capillary barrier [18], [19]. EPCs may be essential for lung repair following injury and may represent a pool of cells able to restore endothelial integrity at sites of endothelial denudation [6], [20]. Previous studies showed that patients with bacterial pneumonia who present reduced EPCs levels tend to have persistent fibrotic changes following recovery from the disease [6]. However, data in respect of the role of EPCs in other lung diseases, such as IPF, are sparse. Fadini et al [21] studied EPCs in a “mixed” population which included several obstructive and restrictive lung disorders. They reported reduced EPCs in restrictive disorders (sarcoidosis, fibrothorax, IPF, etc) as compared to obstructive patients and controls. The same team have previously reported reduced EPCs in patients with IPF associated pulmonary hypertension [22]. Though further studies are necessary in order to evaluate the specific role of EPCs in IPF, our findings taken together with Fadini’s data [21], [22] may suggest that inadequate levels of EPCs may potentially contribute to suppressed repair and recovery of the damaged endothelium within the IPF lung and thereby may drive the sequence of events in profibrogenic direction. On the basis of this conception one may speculate that an endothelial pathway may be involved in the multiple mechanisms responsible for IPF pathogenesis. However, due to the cross-sectional nature of the study our findings cannot establish causality. Therefore, the reduced early EPCs may be an epiphenomenon of the disease and may result from increased consumption and exhaustion of early EPCs due to injury of the pulmonary vessel wall. Clearly, further studies are warranted in order to clarify the exact role of early EPCs in IPF.

A plausible explanation of reduced levels of early EPCs could be either due to reduced production of EPCs from the bone marrow or due to reduced migration after stimulus. However, IPF lacks systemic manifestations and is almost universally limited to the lung. In the present study, bone marrow sampling has not been performed. Nevertheless, blood cell examination was normal in our patients and patients under potentially cytotoxic treatment have been excluded from the study. On the other hand, one should note that VEGF levels in IPF patients were similar to those in control subjects in the present study. In this respect, our data are not suggestive of reduced migration of EPCs due to low VEGF levels. We certainly acknowledge that a defect in other molecular signals that induce the release of early EPCs in the peripheral circulation resulting in the reduction of circulating EPCs levels, cannot be excluded.

In the present study, we investigated the relationship between early EPCs cultures and clinical markers of disease severity which reflect hypoxemia such as P_(A-a)_O_2_, and we found that P_(A-a)_O_2_ was inversely correlated with early EPCs levels. One could speculate that chronic hypoxia-mediated stimulation of the bone marrow may lead to exhaustion of the early EPCs pool, thus leading to reduced EPCs levels in the circulation. However, definitive conclusion is hard to be drawn due to limited available published data. *In vitro* experimental studies have documented increased proliferation and differentiation capacity of EPCs cultured under hypoxic conditions [23]. On the other hand, *in vivo* data in restrictive diseases [21] failed to demonstrate any correlation of EPCs levels with markers of hypoxia, such as arterial oxygen tension. Thus, the relationship between hypoxia and early EPCs levels in IPF needs further investigation.

In the present investigation, we observed that IPF patients presented reduced levels of early EPCs and enhanced endogenous VEGF expression. In agreement with our findings, Avouac et al [24] have reported increased VEGF mRNA levels of EPCs following hypoxic exposure. Hypoxic conditions are associated with transcriptional induction of VEGF gene [25] and is expected to be enhanced in chronic lung diseases associated with progressive decline of gas exchange, such as IPF. Enhanced VEGF mRNA production by EPCs may reflect a compensatory mechanism to overcome reduced EPCs levels and retain endothelial homeostasis in the IPF lung.

In agreement with previous reports, some IPF patients presented signs of right cardiac functional alterations suggestive of increased pressures in the pulmonary circulation. Mean sPAP was 39.59±12.35 mmHg in our cohort and approximately 50% of patients presented sPAP≥35 which is considered to be suggestive of PH [16], [17]. Furthermore, we evaluated the relationship between EPCs levels and sPAP and we found that IPF patients with sPAP≥35 mmHg exhibit reduced early EPCs levels when compared to patients with sPAP<35 mmHg. An explanation for this relationship might be that early EPCs depletion triggers the development of PH or pre-PH mechanisms in IPF through endothelial dysfunction. This is consistent with data from clinical observational studies which have shown reduced levels of EPCs in PH [26], [27]. Notably, in animal models of PH, EPCs administration may prevent right ventricular systolic hypertension and may restore pulmonary haemodynamics and improve survival [28]. Furthermore, recent findings suggest that endothelial cell dysfunction may underlie the pathogenesis of IPF-associated PH [29], [30]. Our findings support the latter hypothesis.

In the present study we used echocardiography in order to examine right heart involvement in our patients. Echocardiograpy is not the gold standard for the diagnosis of PH, however it represents a reliable and relatively accurate non-invasive method to evaluate PH with higher rates of compliance for research purposes [14], [31]. In our study we excluded patients with left ventricular systolic dysfunction or known cardiac disease which could increase sPAP due to increased left ventricular (LV) filling pressures. Diastolic function as assessed from transmitral Doppler flow and TDI did not differ between patients with sPAP ≥35 mmHg and those with sPAP<35 mmHg (data not shown) and thus it is not possible that LV diastolic dysfunction contributed to the increase in sPAP due to postcapillary hypertension. We certainly acknowledge that the number of the patients included in our study is relatively small. Due to the strict criteria applied (diabetes, hypertension, etc) we excluded a significant number of subjects from the pool of patients that are regularly followed-up by our insterstitial lung diseases clinic. Additionally we did not observe any significant overlap of EPCs levels in patients and controls. Thus we may speculate that it is unlikely that the enrollment of additional patients would significantly affect our results. However more studies are required in order to confirm our findings.

In conclusion, the present study provides evidence suggesting that early EPCs levels are reduced in IPF patients compared to controls and that EPCs derived from IPF patients, exhibit increased endogenous VEGF production. Notably, early EPCs levels were significantly lower in patients with increased pressure in the pulmonary circulation. Further studies are required in order to investigate the cause(s) of early EPCs depletion and to determine the clinical impact of our observations.
